# 

**Published:** 1899-07

**Authors:** 


### NOTICES.

### National Dental Association Meeting.

At the second annual meeting of the National Dental Association, to be held at Niagara Falls, N. Y., commencing Aug. 1, 1899, the following program will be carried out:

*Tuesday.*—8 A.M.: Meeting of the Executive Committee and Treasurer, for the examination of credentials and receiving of dues. 10 A.M.: Meeting of the sections. 11 A.M.: Association called to order; calling the roll; reading of minutes; miscellaneous business; reports of committees, officers, etc.; president's address; discussion; miscellaneous business. 1 A.M.: Adjournment.

Afternoon session: Meeting of the sections.

Evening session, 7:30 P.M: Reading of minutes; miscellaneous business; reading and discussion of reports and papers of the sections, commencing with Section II., S. H. Guilford, chairman, M. F. Finley, secretary; miscellaneous business. Adjournment.

*Wednesday.*—9 A.M.: Reading of minutes; miscellaneous business; reports, papers and discussion. 12:30 P.M.: Organization of sections; miscellaneous business. Adjournment.

Evening session, 7:30 P.M.: Reading of minutes; reports, papers and discussion. Adjournment.

*Thursday.*—9 A.M.: Reading of minutes; miscellaneous business; reports, papers and discussion. 1 P.M: Adjournment.

Evening session, 7:30 P.M.: Reading of minutes; reports, papers and discussion. Adjournment.

*Friday.*—9 A.M.: Reading of minutes; miscellaneous business; reports, papers and discussion. 11:30 P.M: Selection of place of next meeting, and election of officers. Adjournment till 3 P.M.

Afternoon session, 3 P.M.: Reading of minutes; miscellaneous business; reports, papers and discussion; miscellaneous business; reading of minutes; installation of officers; miscellaneous business; final reading of minutes. Final adjournment.

The sections will be called in the following order, and any section failing to respond will be passed and not again called until all the others have been:

Section II—Dental Education, Literature and Nomenclature. S. H. Guilford, chairman; M. F. Finley, sec'y.

Section III—Operative Dentistry. J. Y. Crawford, chairman; Frank Holland, sec'y.

Section IV—Histology and Microscopy. T. L. James, chairman; L. L. Dunbar, sec'y.

Section V—Materia Medica and Therapeutics. J. S. Cassidy, chairman; A. W. Harlan, sec'y.

Section VI—Physiology and Etiology. J. D. Patterson, chairman; L. E. Custer, sec'y.

Section VII—Anatomy, Pathology and Surgery. W. C. Barrett, chairman; W. F. Lewis, sec'y.

Section VIII—Hygiene and Prophylactic Dentistry. J. Taft, chairman; H. B McFadden, sec'y.

Section IX—Orthodontia. V. H. Jackson, chairman; C. L. Goddard, sec'y.

Section X—Clinics. H. J. McKellops, chairman; C. L. Culver, sec'y.

Section I—Prosthetic Dentistry, Chemistry and Metallurgy. I. N. Broomell, chairman; Wm. Ernest Walker, sec'y.

Addrcsses will be delivered by L. E. Custer, Dayton, Ohio, on "Electricity." T. W. Brophy, Chicago, and Thos. Fillebrown, Boston, on "Surgical Treatment of Cleft Palate."

As it is necessary for the sections to have their meetings and pass upon all papers before they are presented to the general body, we have not attempted to arrange the program for each, simply announcing the sections in the order in which they will be called. Following is a list of the papers promised: "Bacteriology" and "Plain Words with My Critics," J. Leon Williams, London; "Susceptibility and Immunity to Dental Caries," G. V. Black, Chicago; "The Abuse of Crown and Bridge Work," W. George Beers, Montreal; "Porcelain Enamel Inlays," Dr. N. S. Jenkins, Dresden; "Orthodontia" (illustrated), Dr. Edward H. Angle, St. Louis; "The Absolute Efficiency of the Controllers on the Market for Dental Cataphoresis," Dr. W. A. Price, Cleveland; "Dental Electricity," Dr. L. E. Custer, Dayton; "The Practical Side of It," Dr. S. S. Stowell, Pittsfield; "A Bastard Profession," Dr. E. P. Beadles, Danville; "Cements," Dr. E. K. Wedelstaedt, Minneapolis; "The Reflexes of the Three Lower Molars," Dr. James Truman, Philadelphia; "Gomphosis," Dr. B. H. Catching, Atlanta; "Prognathism, Extraction and Delay versus Expansion and Early

Attention" (illustrated), Dr. R. Ottolengui, New York; "Some Phases of the Cement Question," Dr. W. V.-B. Ames, Chicago; "A Study of Hare-Lip and Cleft Palate" (illustrated), Dr. Thomas Fillebrown, Boston; "Dies and Counter-Dies," Dr. Robert H. Nones, Philadelphia; "Constitutional Deterioration the Cause of Dental Caries," Dr. Harvey, Battle Creek; "Oral Affections in Secondary Syphilis" (illustrated), Dr. W. C. Barrett, Buffalo; "The Physiological Relation of the Adult Tooth-Pulp to the Economy," Dr. C. L. Hungerford, Kansas City; "Etiology of Gnathic Abnormalities," Dr. A. H. Thompson, Topeka; "Some New Points in the Anatomy of the Face and Jaws" (illustrated), Dr. M. H. Cryer, Philadelphia; "The Present Status of Continuous Gum," D. D. Smith, Philadelphia; "Impressions and Models," C. D. Lukens, St. Louis; "Literary Requirements of Dental Students," Wm. Crenshaw, Atlanta; "Dental Articulation and Occlusion," Wm. Ernest Walker, Pass Christian, Miss.; "Some Phases of the Dental Educational Problem," C. S. Butler, Buffalo; "Care of Children's Teeth," C. N. Johnson, Chicago; "A Review of Operative Dentistry in One Man's Lifetime," J. N. Crouse, Chicago; "Choice of Filling Materials for the Teeth between the Ages of Eight and Eighteen," S. B. Palmer, Syracuse; "Filling Superior Oral Teeth," D. D. Smith, Philadelphia; "The Text-Book and the Teacher," W. Storer How, Philadelphia; "Report of Therapeutic Congress," J. S. Cassidy, Covington; "Recent Advances in Therapeutics," A. W. Harlan, Chicago; "Counter-Irritation," W. E. Griswold, Denver; "The Radical Cure of Congenital Cleft Palate" (illustrated by cases in practice), T. W. Brophy, Chicago; "The Pathology of the Pericemental Membrane," M. L. Rhein, New York City; "Roentgen Rays," C. Edmund Kells, New Orleans; "The Results that Follow the Extraction of Permanent Teeth," E. A. Bogue, New York City; "The Significance of Neuro-Dynamics in Oral Diseases," G. V. I. Brown, Milwaukee; "Why Dentists Should Recommend the Use of Brush and Water," Laurence Leonard, Waseca, Minn.; "Models and Appliances," T. P. Hinman, Atlanta; "Classification and Therapeutics of the Essential

Oils," A. H. Peck, Chicago; "The True Function of Saliva," L. M. Cowardin, Richmond; "The Special Preliminary Education of the Operative Dentist," R. H. Hofheinz, Rochester; "The Uses and Limitations of Formaldehyd in Dentistry," F. W. Low, Buffalo; "Clinical Proof that the Dentin can be Nourished after the Pulp is Destroyed," Joseph Head, Philadelphia; "Porcelain Inlays," B. C. Russell, Keene, N. H.; "The Mixter-Smith Method of Reducing Dislocation of the Lower Jaw," M. C. Smith, Lynn, Mass.; "Gold versus Amalgam," H. H. Johnson, Macon, Ga.; "Painless Dental Operations," B. Holly Smith, Baltimore; "The Hygiene of the Dental Office," I. P. Wilson, Burlington, Iowa; "Dental Hygiene as Applied to Public Schools," E. F. Adair, Harmony Grove, Ga.; "Hygiene," J. A. Chapple, Atlanta, Ga.; "A Criticism on Pyorrhea Alveolaris," S. Freeman, New York.

OFFICERS.

President, H. J. Burkhart, Batavia, N. Y.; vice-president for East, S. H. Guilford, Philadelphia, Pa.; vice-president for West, Thomas E. Weeks, Minneapolis, Minn.; vice-president for South, B. Holly Smith, Baltimore, Md.; recording secretary, Geo. H. Cushing, Burbank, Cal.; ass't secretary, Wm. E. Walker, Pass Christian, Miss.; corresponding secretary, Emma E. Chase, St. Louis, Mo.; treasurer, H. W. Morgan, Nashville, Tenn.

EXECUTIVE COMMITTEE FOR SESSION OF 1899.

J. N. Crouse, chairman; M. F. Finley, sec'y. First Division, Committee of Arrangements: J. N. Crouse, L. G. Noel, H. A. Smith. Second Division, Credentials and Auditing Committee: V. H. Jackson, J. D. Patterson, M. F. Finley. Third Division, Committee on Voluntary Essays: C. S. Butler, G. V. I. Brown, J. Y. Crawford.

A railroad rate of one and one-third fare on the certificate plan will be obtained, also reduced rates on C. & B. and Northern Transportation steamship lines. It is suggested that members living at a considerable distance organize parties and thereby be enabled to secure lower rates from railroad companies.

Following is a list of hotels and rates per day: Cataract House, $3 to $4; International Hotel, $3 to $4; Kaltenback

Hotel, $3; Imperial Hotel, $2.50 to $4; Columbia Hotel, $1.50 to $2; Temperance House, $1.50 to $2; Niagara Falls House, $2; Niagara House, $2.

Dr. M. O. Cooley, of Niagara Falls, N. Y., will engage rooms and answer any questions regarding local arrangements for the meeting. Definite meeting-places for sections will be announced later.

It is the wish of the officers of the Association that members make special efforts to be present at section meetings, on account of the unusual number of valuable papers which must first be passed upon by the section to which they properly belong.

### Twenty-ninth Annual Session of the New Jersey State Dental Society.

The twenty-ninth annual session of the New Jersey State Dental Society, will be held in the Auditorium at Asbury Park, at 10 A. M., July 19, 20, 21, 1899. This is one of the oldest and most progressive State dental societies in the country. It is to be held this year at one of the most beautiful and pleasant places imaginable. Very few places are as pleasant and attractive as Asbury Park.

The program is a very attractive one and will doubtless be carried out to the letter. Whoever has the opportunity and privilege of being present at these meetings will be doubly blest.

There is no doubt but there will be a large attendance and a very profitable meeting had, so it is hoped that everybody within reach will be present.

